# Adaptive Finite Element Model for Simulating Crack Growth in the Presence of Holes

**DOI:** 10.3390/ma14185224

**Published:** 2021-09-10

**Authors:** Abdulnaser M. Alshoaibi, Yahya Ali Fageehi

**Affiliations:** Mechanical Engineering Department, Jazan University, P.O. Box 114, Jazan 45142, Saudi Arabia; yfageehi@jazanu.edu.sa

**Keywords:** fatigue crack growth, adaptive mesh, stress intensity factors, fatigue life, finite element method, holes

## Abstract

This study presents a developed finite element code written by Visual Fortran to computationally model fatigue crack growth (FCG) in arbitrary 2D structures with constant amplitude loading, using the linear elastic fracture mechanics (LEFM) concept. Accordingly, optimizing an FCG analysis, it is necessary to describe all the characteristics of the 2D model of the cracked component, including loads, support conditions, and material characteristics. The advancing front method has been used to generate the finite element mesh. The equivalent stress intensity factor was used as the onset criteria of crack propagation, since it is the main significant parameter that must be precisely predicted. As such, a criterion premised on direction (maximum circumferential stress theory) was implemented. After pre-processing, the analysis continues with incremental analysis of the crack growth, which is discretized into short straight segments. The adaptive mesh finite element method was used to perform the stress analysis for each increment. The displacement extrapolation technique was employed at each crack extension increment to compute the SIFs, which are then assessed by the maximum circumferential stress theory to determine the direction of the crack growth and predict the fatigue life as a function of crack length using a modified form of Paris’ law. The application examples demonstrate the developed program’s capability and performance.

## 1. Introduction

Fracture mechanics’ main goal is to figure out how quickly a crack’s shape changes. Will it grow and if it grows, at what rate and into what configuration under certain loadings and conditions? The computing requirements corresponded to obtaining the components stress, strain, energy, and displacement, which might extract the driving force for crack propagation. In recent decades, computational fracture mechanics has progressed significantly, with a variety of new methods for stress evaluation enabling complex fracture mechanics’ difficulties to be evaluated at a low computational cost [1]. To evaluate the FCG in metallic and aircraft structures, conventional methods rely on LEFM to compute the fatigue life of these components under different loading and boundary conditions [2].

Fatigue represents the most common phenomenon of catastrophic structural failure on mechanical structures and systems. Researchers have been striving to understand the fatigue damage processes in materials subject to cyclic loads for many of the last decades. Fatigue cracks in structural components, pipelines, aircraft fuselages, ships, marine structures, and other similar structures may cause significant damage and even catastrophic failure. However, detection of a crack does not necessarily imply that the service life has ended. Fatigue cracks in redundant structures often show retarded growth under moderate stress, allowing part of the crack growth to be included in the range of reasonable service life, but only if crack growth can be accurately estimated, monitored, and affected details fixed before the beginning of a critical structural situation. The potential of incorporating a portion of crack extensions in the service life of components provides the foundation for the development of the design principle known as “damage tolerant design,” which relates to components that are designed to operate with fatigue damage in a permissible limit [3].

The LEFM techniques have been extensively accepted for use on long cracks under small-scale yielding circumstances at the crack tip, i.e., the Paris regime [4,5]. The stress intensity factors (SIFS) are one of the most dominant parameters in fracture mechanics, since they are used in predicting both initiation and propagation of crack trajectory. The magnitude of the crack tip singularity and condition are defined by SIFs; hence, the stresses all over it increase proportionally to SIFs; if known, all components of stress, strain, and displacement can be calculated. There are numerous stress intensity factors estimation handbooks for distinct geometries and loads [6,7,8,9]. These numerical solutions cover a variety of geometries and loading conditions that are essential in predicting the structural failure of cracked bodies. Over 600 formulae for estimating SIFs values for various crack configurations, body geometries, and certain loading conditions are reported. The limitations of the analytical solution of SIFs suggest a numerical analysis approach to fracture problems in engineering practice, as it is almost impossible to prevent the occurrence of cracks in the structure, which may be caused by: production method, heat treatments of metals, transport, etc. The specific option is to analyze the characteristics of these cracks to assess the component life of the service (i.e., the critical size of the crack or a confident fatigue cycles number). Experimental tests and inspections may be carried out, but this late alternative is costly. Another alternative is to switch to numerical models due to the problem’s complexity, since there are certain cases where the experimental setup is too complicated to be viable. Over the years, several works for using numerical techniques have been developed: Finite Element Method (FEM), Discrete Element Method (DEM) [10,11,12], the Element Free Galerkin (EFG) method [13], Extended Finite Element Method (XFEM) [14,15], cohesive element method [16], and phase-field method [17]. Most problems in crack propagation involving mixed-mode requires predicting crack path and growth while updating the model as the geometry changes; several studies predict crack growth with a high degree of precision [18,19]. The FEM is a comprehensive numerical tool used in engineering applications to predict the behavior of complicated geometries and structures. Therefore, the FEM analysis seems to be a numerical approach used in the propagation of fatigue cracks. Two categories of numerical methods mostly used for computing SIFs are: (1) displacement matching, such as displacement extrapolation methods [20]; and (2) energy-based approaches, which include the crack closure integral method [21,22], the J-integral technique [23], and so on. The most popular approach is the displacement extrapolation method [24].

The developed source code is software that provides results comparable to those achieved with the commercial software commonly available for the study of fracture mechanics. The characterization of stress and displacement fields, which is necessary for the estimation of stress intensity factors, has the greatest computational cost in the analysis; evaluating large structures requires regular computers with present computational capabilities. Commercial software may also be used to model fracture propagation and fatigue life prediction; however, such software is highly expansive, and it is practically difficult to access the source code for further development. The proposed program’s effectiveness was shown in a variety of scenarios with accurate results e.g., [18,25,26,27,28].

## 2. Developed Program Procedure

**Figure 1:**
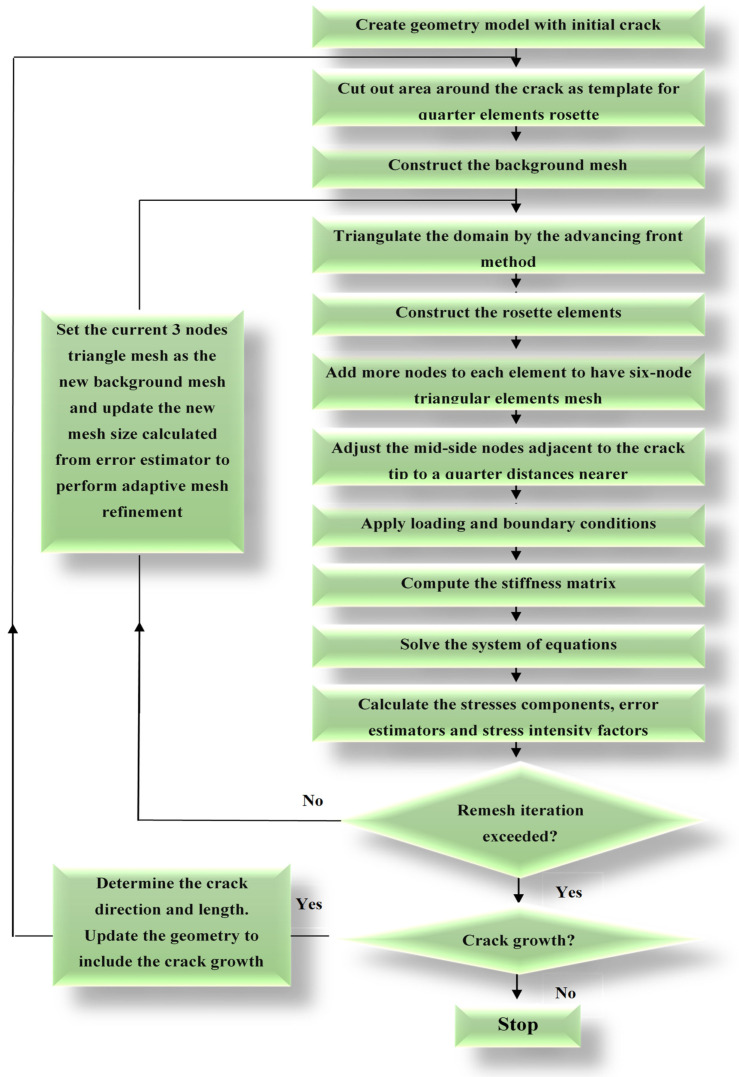
General flowchart of the program.

Pre-processing, processing, and post-processing are primary components of the finite element procedure. The finite element processing is the most prolonged part of the estimation from observation; it includes the calculation and assembly methods of stiffness matrices equations and the solver system. The proposed software is a finite element simulation procedure for analysis of two-dimensional crack growth procedures under linear elastic fracture mechanics assumption. This developed code estimates the 2D quasi-static crack growth considering the mechanical boundary conditions of the fracture. For the adaptive mesh FE analysis, four primary aspects are used: the mesh generation method, the crack criterion, the crack growth criterion, and the crack propagation methodology. The mesh refinement can be controlled by the characteristic scale of each element, which is predicted by the error estimator. The solution errors are computed after each load stage is completed. The incremental analysis is interrupted, and a new FE model is generated when the error approaches a specified cumulative error at some point during the procedure. Under the existing boundary conditions, the framework refines the mesh as required. After the new mesh is generated, the solution variables (displacement, stresses, strains, and so on) are mapped into the new mesh from the old. The analysis is restarted and continued until the errors became greater than the pre-decided number. The SIFs are commonly used as a fracture criterion in LEFM. In the FE technique, the directional criteria are the third component of a crack growth simulation using an adaptive mesh. Various techniques are used to estimate the directions of a crack: maximum circumferential stress theory, maximum energy release theory, and the theory of minimum strain energy density. At each stage of crack growth, an FE model is defined. The model is provided as an input for the simulation in the first step. Then, the models given in previous stages are used to produce the algorithm’s output. The elements within the geometry are deleted and reconstructed using an adaptive approach at each step as the crack grows, and the geometry is updated for next propagation process. During crack propagation simulation, an automated adaptive mesh is generated around crack front nodes and in elements that typify higher stress distribution. The finite element mesh is generated using the advancing front method; the generation of framework mesh and development of singular elements have been introduced to the developed software to fulfill fracture analysis criterion. The computational procedure for modeling fatigue crack growth is depicted in Figure 1. The details of these steps are explained by [25,26,28,29,30,31].

### 2.1. Adaptive Mesh Refinement

Adaptive mesh refinement is an optimization method used in the field of FE mesh. In general, an adaptive approach consists of two main components: an experimental error estimation and the node refinement process [32]. The local and global approximation errors can be measured by a posteriori error estimation, while the node refinement process decides whether a refinement is needed or not according to the error data. The majority of error estimators in the FEM framework are classified as either recovery-based error estimators or residual-based error estimators [33]. The employment of recovery methods in the computation of a posteriori error estimators that are used in the present study is one of the most important applications. The errors were evaluated using the recovered solutions by substituting recovered values for exact values, with accuracy exceeding direct finite element solution. Using *h*-refinement type adaptive mesh optimization, the ratio of element norm stress error to average standard stress error for the entire region was determined. This way, each element mesh size is given as:

(1) $$h_{e}=\sqrt{2A_{e}}$$

where *A_e_* is the triangle element area.

The same type of elements is used in the *h*-refinement type but with changes in sizes at some location (larger and smaller) to optimize economy in attaining the desired outcome, based on which a new element size is predicted in the entire domain, hence generating new mesh entirely. Each element norm stress error is represented by:

(2) $$\begin{array}{l} {\left\|e\right\|}_{e}^{2}={\int_{\Omega^{e}}\left(\sigma -\sigma^{*}\right)}^{T}\left(\sigma -\sigma^{*}\right)d\Omega \\ \;\;\;\;\;\;={\int_{\Omega^{e}}\left(\left\{\begin{array}{c} \sigma_{x}\\ \sigma_{y}\\ \tau_{xy}\\ \sigma_{z} \end{array}\right\}-\left\{\begin{array}{c} \sigma_{x}^{*}\\ \sigma_{y}^{*}\\ \tau_{xy}^{*}\\ \sigma_{z}^{*} \end{array}\right\}\right)}^{T}\left(\left\{\begin{array}{c} \sigma_{x}\\ \sigma_{y}\\ \tau_{xy}\\ \sigma_{z} \end{array}\right\}-\left\{\begin{array}{c} \sigma_{x}^{*}\\ \sigma_{y}^{*}\\ \tau_{xy}^{*}\\ \sigma_{z}^{*} \end{array}\right\}\right)d\Omega \end{array}$$

However, the mean norm stress error in the entire domain Ω is given as:

(3) $$\begin{array}{l} {\left\|\hat{e}\right\|}^{2}=\frac{1}{m}\sum_{e=1}^{m}\int_{\Omega^{e}}\sigma^{T}\sigma d\Omega \\ \;\;\;\;\;\;\;=\frac{1}{m}\sum_{e=1}^{m}\int_{\Omega^{e}}{\left\{\begin{array}{c} \sigma_{x}\\ \sigma_{y}\\ \tau_{xy}\\ \sigma_{z} \end{array}\right\}}^{T}\left\{\begin{array}{c} \sigma_{x}\\ \sigma_{y}\\ \tau_{xy}\\ \sigma_{z} \end{array}\right\}d\Omega \end{array}$$

where *m* is the total number of elements in the whole domain, and Ω and σ* are the smoothed stress vector. A quadratic triangle element with six nodes is used in this study.

**Figure 2:**
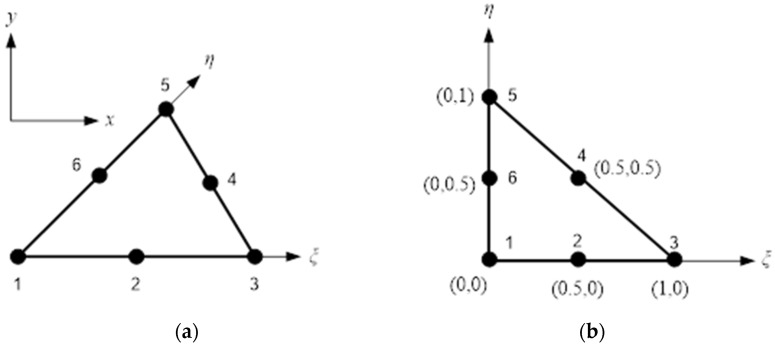
A quadratic triangle element in (**a**) global coordinates system and in (**b**) a natural coordinates system.

Figure 2 depicts this element in the global coordinates system (*x, y*) and its representation in the natural coordinates system (ξ,η).

The summation of quadratics according to the Gauss–Radau rules will be used to convert the integration with the triangular isoparametric dimension in the FEM as follows:

(4) $$\begin{array}{l} {\left\|e\right\|}_{e}^{2}\;={\int_{-1}^{1}\int_{-1}^{1}\left(\left\{\begin{array}{c} \sigma {\left(\xi ,\eta \right)}_{x}\\ \sigma {\left(\xi ,\eta \right)}_{y}\\ \tau {\left(\xi ,\eta \right)}_{xy}\\ \sigma {\left(\xi ,\eta \right)}_{z} \end{array}\right\}-\left\{\begin{array}{c} \sigma {\left(\xi ,\eta \right)}_{x}^{*}\\ \sigma {\left(\xi ,\eta \right)}_{y}^{*}\\ \tau {\left(\xi ,\eta \right)}_{xy}^{*}\\ \sigma {\left(\xi ,\eta \right)}_{z}^{*} \end{array}\right\}\right)}^{T}\left(\left\{\begin{array}{c} \sigma {\left(\xi ,\eta \right)}_{x}\\ \sigma {\left(\xi ,\eta \right)}_{y}\\ \tau {\left(\xi ,\eta \right)}_{xy}\\ \sigma {\left(\xi ,\eta \right)}_{z} \end{array}\right\}-\left\{\begin{array}{c} \sigma {\left(\xi ,\eta \right)}_{x}^{*}\\ \sigma {\left(\xi ,\eta \right)}_{y}^{*}\\ \tau {\left(\xi ,\eta \right)}_{xy}^{*}\\ \sigma {\left(\xi ,\eta \right)}_{z}^{*} \end{array}\right\}\right)t^{e}\det J^{e}d\xi d\eta \\ \;\;\;\;\;\;\;={\sum_{p=1}^{3}\left(\left\{\begin{array}{c} \sigma {\left(\xi_{p},\eta_{p}\right)}_{x}\\ \sigma {\left(\xi_{p},\eta_{p}\right)}_{y}\\ \tau {\left(\xi_{p},\eta_{p}\right)}_{xy}\\ \sigma {\left(\xi_{p},\eta_{p}\right)}_{z} \end{array}\right\}-\left\{\begin{array}{c} \sigma {\left(\xi_{p},\eta_{p}\right)}_{x}^{*}\\ \sigma {\left(\xi_{p},\eta_{p}\right)}_{y}^{*}\\ \tau {\left(\xi_{p},\eta_{p}\right)}_{xy}^{*}\\ \sigma {\left(\xi_{p},\eta_{p}\right)}_{z}^{*} \end{array}\right\}\right)}^{T}\;\left(\left\{\begin{array}{c} \sigma {\left(\xi_{p},\eta_{p}\right)}_{x}\\ \sigma {\left(\xi_{p},\eta_{p}\right)}_{y}\\ \tau {\left(\xi_{p},\eta_{p}\right)}_{xy}\\ \sigma {\left(\xi_{p},\eta_{p}\right)}_{z} \end{array}\right\}-\left\{\begin{array}{c} \sigma {\left(\xi_{p},\eta_{p}\right)}_{x}^{*}\\ \sigma {\left(\xi_{p},\eta_{p}\right)}_{y}^{*}\\ \tau {\left(\xi_{p},\eta_{p}\right)}_{xy}^{*}\\ \sigma {\left(\xi_{p},\eta_{p}\right)}_{z}^{*} \end{array}\right\}\right)t^{e}\det J^{e}W_{p} \end{array}$$

and similarly

(5) $${\left\|\hat{e}\right\|}^{2}=\frac{1}{m}\sum_{e=1}^{m}{\sum_{p=1}^{3}\left(\left\{\begin{array}{c} \sigma {\left(\xi_{p},\eta_{p}\right)}_{x}\\ \sigma {\left(\xi_{p},\eta_{p}\right)}_{y}\\ \tau {\left(\xi_{p},\eta_{p}\right)}_{xy}\\ \sigma {\left(\xi_{p},\eta_{p}\right)}_{z} \end{array}\right\}\right)}^{T}\;\left(\left\{\begin{array}{c} \sigma {\left(\xi_{p},\eta_{p}\right)}_{x}\\ \sigma {\left(\xi_{p},\eta_{p}\right)}_{y}\\ \tau {\left(\xi_{p},\eta_{p}\right)}_{xy}\\ \sigma {\left(\xi_{p},\eta_{p}\right)}_{z} \end{array}\right\}\right)t^{e}\det J^{e}W_{p}$$

where $t^{e}$ is the element thickness for a plane stress condition, $t^{e}=1$ is the element thickness for the plane strain condition, *W_P_* is a weighting factor, and $J^{e}$ is the Jacobian matrix, which was defined as:

(6) $$J^{e}\;=\;\left[\begin{array}{cc} \frac{\partial x}{\partial \xi } & \frac{\partial y}{\partial \xi }\\ \frac{\partial x}{\partial \eta } & \frac{\partial y}{\partial \eta } \end{array}\right]\;=\;\left[\begin{array}{cc} \sum_{i=1}^{r}\frac{\partial N_{i}^{e}}{\partial \xi }\;x_{i}^{e} & \sum_{i=1}^{r}\frac{\partial N_{i}^{e}}{\partial \xi }\;y_{i}^{e}\\ \sum_{i=1}^{r}\frac{\partial N_{i}^{e}}{\partial \eta }\;x_{i}^{e} & \sum_{i=1}^{r}\frac{\partial N_{i}^{e}}{\partial \eta }\;y_{i}^{e} \end{array}\right].$$

Subsequently, the relative stress norm error $\xi_{e}$ for each item is substantially less than 5%, which is conventional for many applications in engineering. Thus,

(7) $$\zeta_{e}=\frac{{\left\|e\right\|}_{e}}{\left\|\hat{e}\right\|}\leq \zeta$$

and the relative stress error level of the new element is identified as a permissible error by:

(8) $$\varepsilon_{e}=\frac{{\left\|e\right\|}_{e}}{\zeta \left\|\hat{e}\right\|}\leq 1.$$

This necessitates the refinement of each element with $\varepsilon_{e}$ > 1, together with the anticipation of a new mesh size. Asymptotic convergence rate criteria have been used in this case, which assumes:

(9) $${\left\|e\right\|}_{e}\propto h_{e}^{p}$$

where *p* denotes polynomial order approximation, which was selected as *p* = 2 in the for the quadratic polynomial. Thus, the approximate size of the new element is:

(10) $$h_{N}=\frac{1}{\sqrt{\varepsilon_{e}}}h_{e}.$$

Premised on the specified quantity of mesh refinement, the current mesh will be considered as a new background mesh and the advancing front method will be replicated.

### 2.2. Mesh Smoothing

To improve the form of the elements, mesh smoothing is used on completion of its generation process; during this smoothing process, its topological structure is preserved, i.e., the element’s nodal connections are not changed, but inner nodes are relocated to create triangles with mostly better forms. The most effective smoothing technique is the well-known Laplacian smoothing [34]; in terms of computing efficiency, it repositions the inner node to the centroid of the polygon in adjacent nodes. Thus, the new location of internal node *i* is computed:

(11) $$\left(x_{i},y_{i}\right)\;=\;\frac{1}{N_{n}}\;\sum_{j=1}^{N}\left(x_{j},y_{j}\right)\;$$

**Figure 3:**
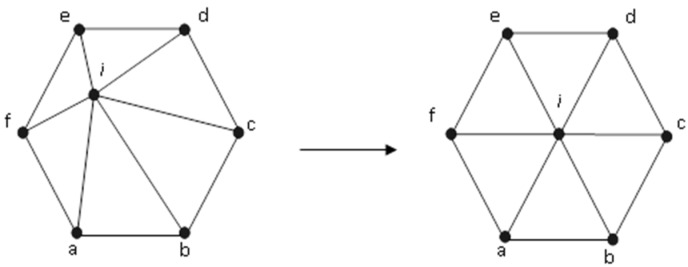
Laplacian smoothing, repositioned of node *i*.

*N* denotes the number of connected nodes to node *i*. There are numerous iterations in the mesh smoothing technique. As seen in Figure 3, the technique is effective, as it modifies the shape of the mesh elements into a better one.

### 2.3. Crack Growth Increment

The incremental crack growth length Δ*a* is at 10–20% of the original crack length *a*, which was more suitable for the smoothed curvature crack growth path in the mixed-mode loading; it is indirectly proportional to the stress intensity ratio (*K_II_/K_I_*). When *K_II_* is relatively large compared to *K_I_*, it indicates a mixed mode ratio and thus smaller incremental length usage to properly justify the smooth crack growth path trajectory. As a result, the Lagrange interpolation approximates the crack length increment as:

(12) $$\Delta a=\left(\left(1-\left|\frac{K_{II}}{K_{I}}\right|\right)\left(20\%\right)+\left|\frac{K_{II}}{K_{I}}\right|\left(10\%\right)\right)a$$

where *K_I_* and *K_II_* are mode I and mode II of stress intensity factors. However, based on the justification, this percentage range can be modified appropriately, as some other studies reported using a 20–50% range [35,36].

### 2.4. Crack Kinking Criteria

Problems in crack propagation require two major criteria: firstly, the certain crack that propagates, and secondly, the direction it propagates. These criteria are premised on two prerequisites on crack propagation and crack kinking. Direction of the crack is determined by the crack kinking criteria, which is divided into three parts: the first category depends upon the local fields at crack extremity, such as the maximum circumferential stress criterion [37] or the maximum strain criterion [38]; the second category is related to the energy distribution within the cracked component, using a universal method such as the maximum energy release rate criterion [39]; the third category is the minimum strain-energy density theory [40].

The maximum circumferential stress theory has been used in the present study for the calculation of the crack direction angle; it states that for isotropic materials subjected to mixed-mode loading, the crack propagates normal to maximum tangential tensile stress. The tangential stresses in polar coordinates for tensile mode *K_I_* and the in-plane shear mode *K_II_* are given by:

(13) $$\begin{array}{l} \sigma_{r}\;=\;\frac{1}{\sqrt{2\pi r}}\cos(\theta /2)\left(K_{I}[1+\sin^{2}(\theta /2)]+\frac{3}{2}K_{II}\sin\theta -2K_{II}\tan(\theta /2)\right)\\ \sigma_{\theta }=\frac{1}{\sqrt{2\pi r}}\cos(\theta /2)\left[K_{I}\cos^{2}(\theta /2)-\frac{3}{2}K_{II}\sin\theta \right]\\ \tau_{r\theta }=\frac{1}{\sqrt{2\pi r}}\frac{\cos(\theta /2)}{2}\left[K_{I}\sin\theta +K_{II}(3\cos\theta -1)\right]. \end{array}$$

$\sigma_{r}$ denotes the normal stress component in the radial direction, $\sigma_{\theta }$ denotes the normal stress component in the tangential direction, and $\tau_{r\theta }$ denotes the shear stress component. The direction normal to the maximum tangential stress is obtained by solving $d\sigma_{\theta }/d\theta =0$ for *θ*. The nontrivial solution is:

(14) $$K_{I}\sin\theta +K_{II}\left(3\cos\theta -1\right)=0$$

whose solution is:

(15) $$\theta =\pm \cos^{-1}\left\{\frac{3K_{II}^{2}+K_{I}\sqrt{K_{I}^{2}+8K_{II}^{2}}}{K_{I}^{2}+9K_{II}^{2}}\right\}.$$

**Figure 4:**
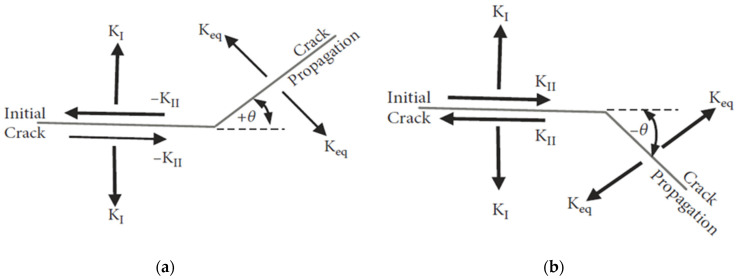
Crack growth angle: (**a**) *K_II_* > 0 and (**b**) *K_II_* < 0.

The sign of *θ* must be reversed to the sign of *K_II_* to guarantee the maximum stress related to crack increment [41]. The two alternatives are seen in Figure 4.

### 2.5. Displacement Extrapolation Technique (DET)

**Figure 5:**
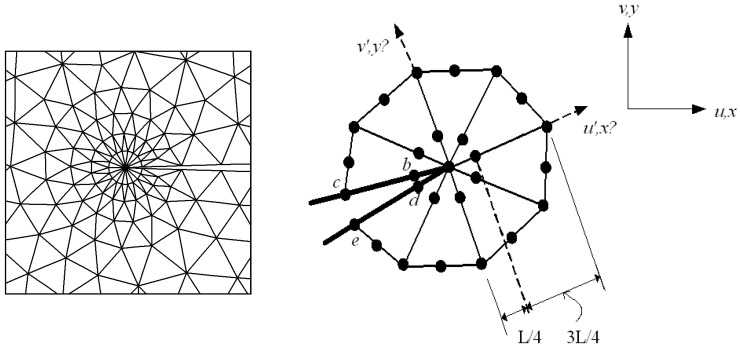
Quarter-point singular elements and coordinates near the crack-tip field [15].

The DET is based on a linear elasticity FEM; it uses triangular quarter-point singular isoparametric elements all over the crack extremity and six-node isoparametric elements elsewhere. In general, the quarter-point element is required to accurately describe the linear-elastic singularity ($1/\sqrt{r}$) for stresses and strains at the crack’s tip. Mid-side nodes are moved next to the crack tip to a quarter-length edge nearer to the crack tip, yielding the polynomial isoparametrically representative of the singularity [42,43]. The natural triangle–quarter-point element is used as crack-tip element, and its configuration follows the schematic creation of the rosette, as shown in Figure 5.

The method of displacement extrapolation [36] was employed to estimate SIFs as:

(16) $$K_{I}=\frac{E}{3(1+\nu )(1+\kappa )}\sqrt{\frac{2\pi }{L}}\left[4({v^{\prime }}_{b}-{v^{\prime }}_{d})-\frac{\left({v^{\prime }}_{c}-{v^{\prime }}_{e}\right)}{2}\right]$$

(17) $$K_{II}=\frac{E}{3(1+\nu )(1+\kappa )}\sqrt{\frac{2\pi }{L}}\left[4({u^{\prime }}_{b}-{u^{\prime }}_{d})-\frac{\left({u^{\prime }}_{c}-{u^{\prime }}_{e}\right)}{2}\right]$$

where *E* is the elasticity modulus, ν is the Poisson’s ratio, κ is the elastic parameter defined as:

(18) $$\kappa \;=\;\{\begin{array}{c} 3-4\nu \;for\;plane\;strain\;\;\\ \frac{\left(3-\nu \right)}{\left(1+\nu \right)}\;for\;plane\;stress\;\;\; \end{array}$$

and *L* denotes the length of the quarter-point element. Where $u^{\prime }$ and $v^{\prime }$ are components of displacement in the *x*′ and *y*′ directions, respectively, as represented in Figure 5.

### 2.6. Static and Fatigue Crack Growth Analysis

All LEFM-based fatigue crack growth prediction models predict the propagation of cracks using numerical integration methods in a cycle-by-cycle (or positive half-cycle) order [44]. The number of fatigue cycles (*N*) represents the damage-elapsed time. This phase, characterized by crack growth per cycle (*da/dN*), is affected by the applied stress intensity factor range. The equivalent stress intensity range at each crack tip must exceed the threshold stress intensity factor to achieve fatigue crack growth, which is described as:

(19) $$\Delta K_{th}=f\Delta \sigma_{th}\sqrt{\pi a}$$

where *f* is both geometry and loading function and $\Delta \sigma_{th}$ denotes the limit range of stress. Equation (19) establishes criteria under which a fatigue crack will not propagate if the applied load is less than the stress range’s limit ($\Delta \sigma <\Delta \sigma_{th}$). However, for fatigue crack propagation, a parameter (equivalent stress intensity factor range, $\Delta K_{Ieq}$) is commonly utilized as an indicator. As a result, fatigue crack propagation will occur if $\Delta K_{Ieq}\;>\Delta K_{th}$; otherwise, there would be no fatigue crack growth. In addition, in static loading, the crack propagates once the equivalent stress intensity factor exceeds the material’s fracture toughness *K_IC_*.

Table 1 lists some of the commonly used models for $\Delta K_{eq}$ proposed by the authors. In the present study, the Tanaka model [45] was implemented in which a modified form of Paris’ law for the relation between crack growth rate and corresponding equivalent stress intensity factor is represented as follows:

(20) $$\frac{da}{dN}=C{\left(\Delta K_{eq}\right)}^{m}$$

where *a* is the crack length, *N* is the number of cycles, *C* is the Paris constant, and *m* is the Paris exponent.

The equivalent stress intensity factor defines the cyclic stresses and stresses ahead of the crack tip and characterizes the rate of crack growth. For a crack increment *da*, Equation (20) can be used to predict the fatigue life cycles as follows:

(21) $$\int_{0}^{\Delta a}\frac{da}{C{\left(\Delta K_{eq}\right)}^{m}}=\int_{0}^{\Delta N}dN=\Delta N$$

## 3. Results and Discussions

### 3.1. Modified Compact Tension Specimen with Different Initial Crack-Tip Position

**Figure 6:**
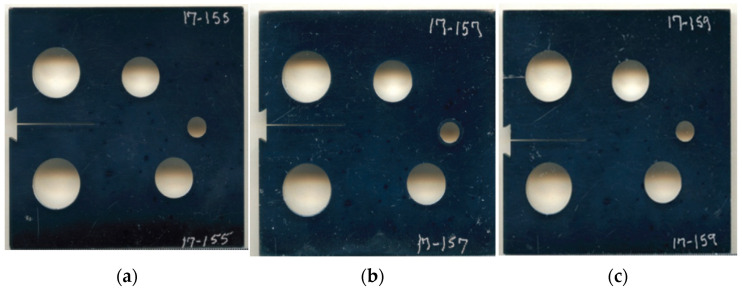
Tested specimens with different notch tip location, (**a**) case 1, (**b**) case 2, and (**c**) case 3.

**Figure 7:**
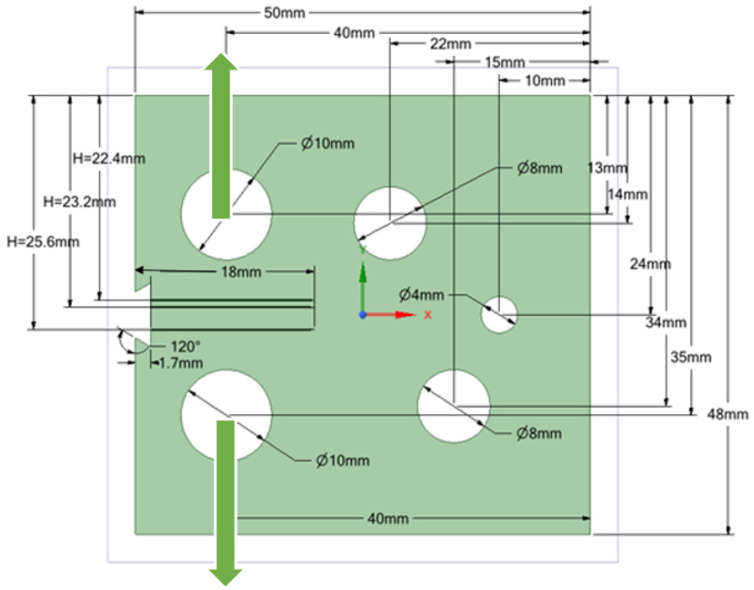
Geometrical dimensions of the Modified Compact tension Specimen (MCTS) with different initial crack-tip position.

**Figure 8:**
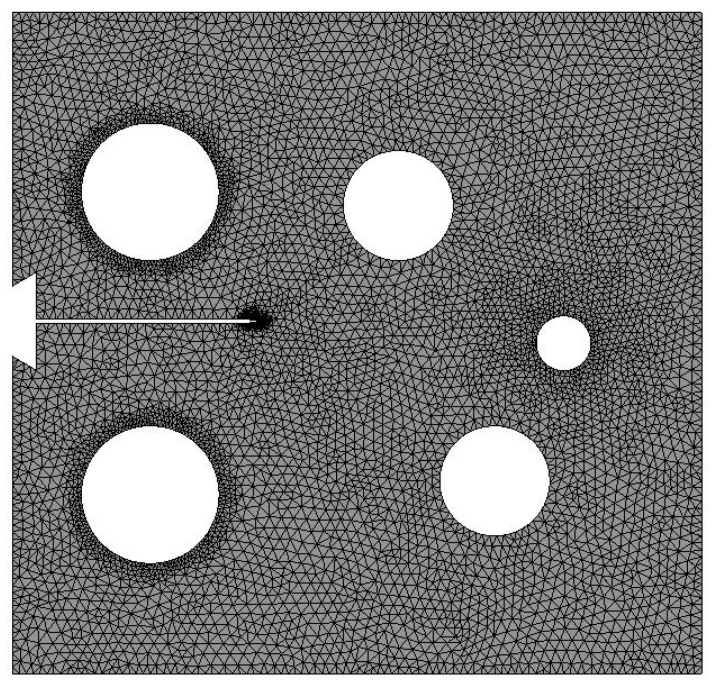
Initial adaptive mesh for the MCTS.

- Three different configurations of the modified compact tension specimen have been investigated in the present study. Wagner [49] conducted the experimental investigation on these specimens, as displayed in Figure 6. The modified specimens differed from standard specimens with three additional holes (Figure 7). These break the symmetry of the standard specimens, yielding curvilinear fatigue crack paths. As illustrated in Figure 7, the vertical notch location (H) is measured with reference to each specimen’s top edge. The notch tip positions in the specimens are represented in Table 2. The initial coordinate for these positions is in the middle of the specimens, beginning from the left-side edge. Depending on the location of the nominal notch, there are three distinct scenarios. Super alloy nickel-based rolled sheets with Young’s modulus *E* = 211 GPa, the Poisson’s ratio *ν* = 0.3, yield stress *σ*_y_ = 422 MPa, and ultimate stress *σ*_u_ = 838 MPa were used for these geometries. A 3.6 kN point load with a load ratio of R = 0.1 was applied. Both the crack-tip path and destination varied with the vertical position of the initial notch (H) (above or below its usual centerline location), as shown in Table 2. The initial adaptive 2D mesh for this geometry is shown in Figure 8 with 190,077 nodes and 120,838 elements.

**Table 2:** Modified Compact tension Specimen (MCTS) notch tip location [50].

Specimen Number	Notch Tip Location (mm)		
	(H)	(x)	(y)
Case 1	22.4	−32	25.6
Case 2	25.6	−32	22.4
Case 3	23.2	−32	24.8

***Case* 1:**

**Figure 9:**
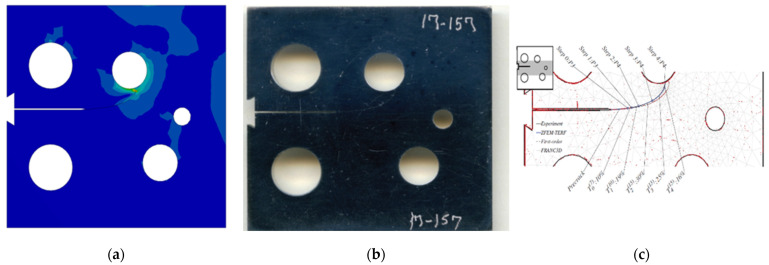
Case 1, predicted crack growth trajectory by (**a**) present study, (**b**) experimental, and (**c**) numerical crack path obtained by [51].

**Figure 10:**
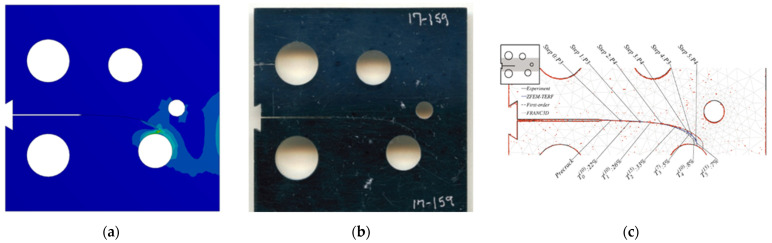
Case 2, predicted crack growth trajectory by (**a**) present study, (**b**) experimental, and (**c**) numerical crack paths obtained by [51].

**Figure 11:**
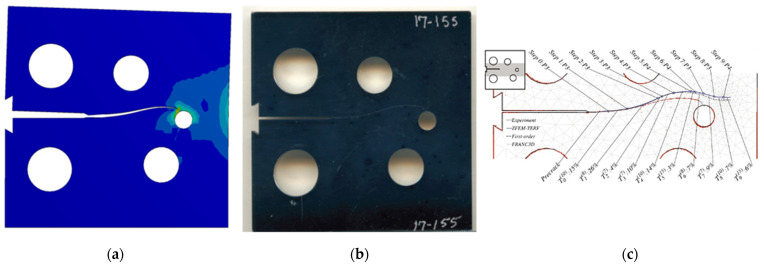
Case 3, predicted crack growth trajectory by (**a**) present study, (**b**) experimental, and (**c**) numerical crack path obtained by [51].

In this case, the initial crack had 22.4 mm height from the top edge of the specimen. Figure 9, Figure 10 and Figure 11 compare the simulated crack growth trajectory as predicted by the developed program to the experimental [49] (Figure 9b) and numerical [51] (Figure 9c). The predicted crack growth paths in the numerical results obtained by [51] were estimated using the following three steps: foremost is the hypercomplex finite element method trial energy response function (ZFEM-TERF) algorithm for crack path prediction; this step uses the trial energy response function (TERF) approach, which is an adaptive progressive fracture approach that adds curvilinear crack path segments to the simulation at each step of the simulation, secondly, they used a finite element model by FRANC3D software, and finally, they used Abaqus software to solve the finite element model produced by FRANC3D. The outcomes of these three steps are shown in Figure 9c; as observed in Figure 9, the predicted crack growth in the present study is almost identical to the experimental path as well as to the three numerical methods results obtained by [51] using the ZFEM-TERF method and FRANC3D.

***Case* 2:**

For the second case, the initial crack has 25.6 mm height from the top edge of the specimen with a 3.2 mm difference of the vertical position. The crack growth path predicted in the present study was in agreement with the experimental path predicted by [49] more than the numerical paths predicted by [51], which has tighter curvature paths as shown in Figure 10.

***Case* 3:**

The third case had an initial crack height of 23.3 mm from the specimen’s top edge; as shown in Figure 11, the predicted crack growth path follows closely the experimental crack predicted by [49] in contrast to the anticipated trajectories from the ZFEM-TERF and FRANC3D simulations performed by [51], which diverged from the experimental trajectory.

**Figure 12:**
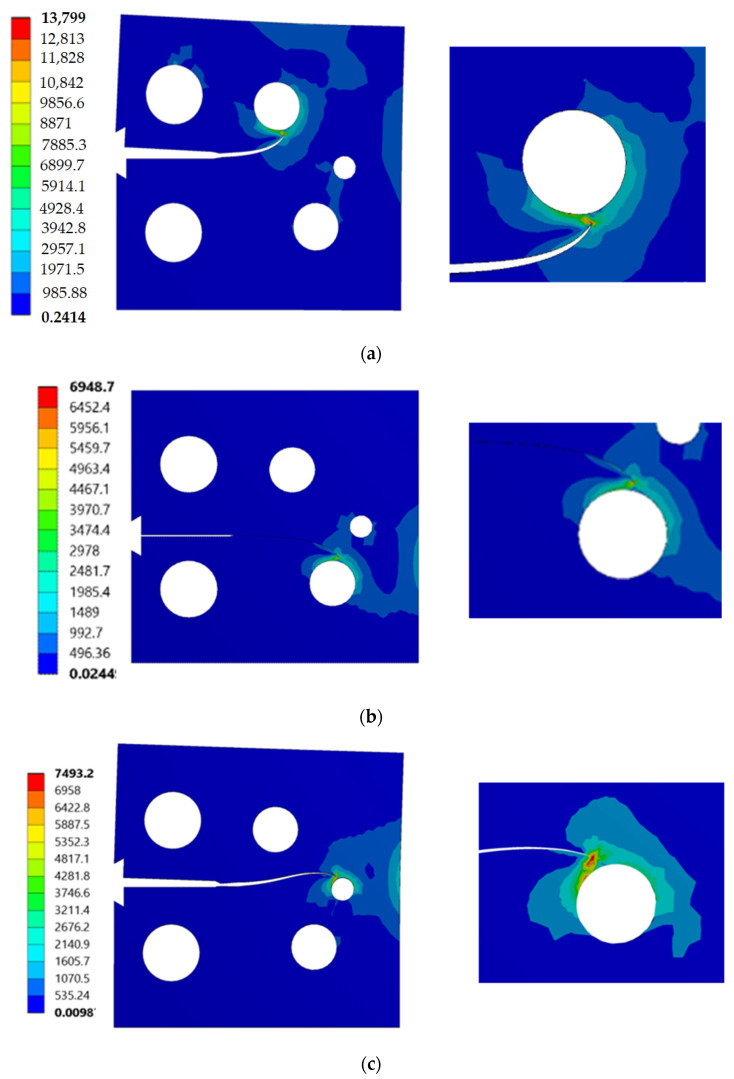
Distribution of von Mises stress (MPa): (**a**) case 1, (**b**) case 2, and (**c**) case 3.

**Figure 13:**
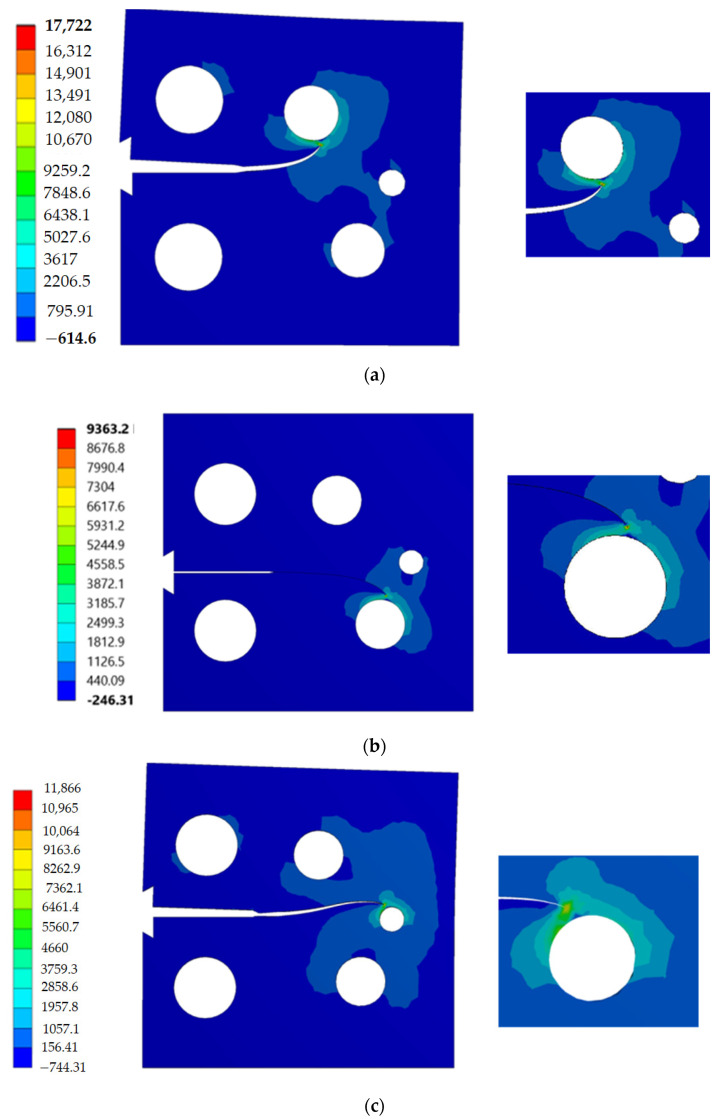
Distribution of maximum principal stress (MPa): (**a**) case 1, (**b**) case 2, and (**c**) case 3.

**Figure 14:**
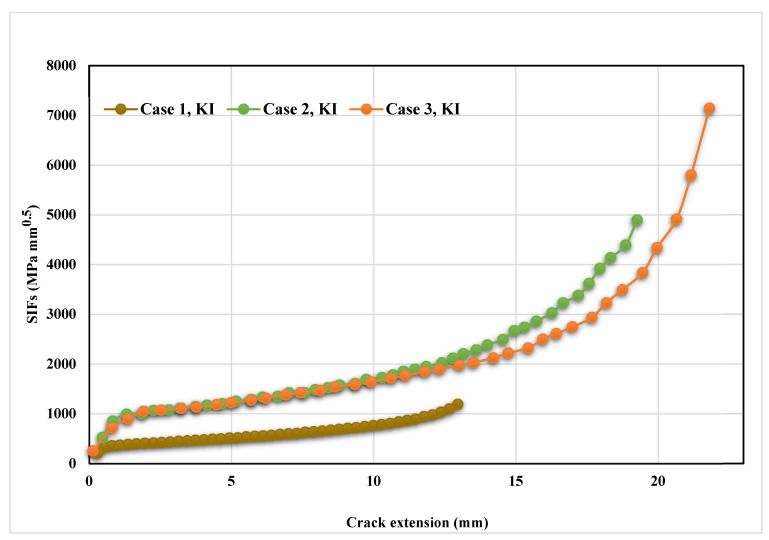
Predicted values of *K_I_* for the three different cases.

**Figure 15:**
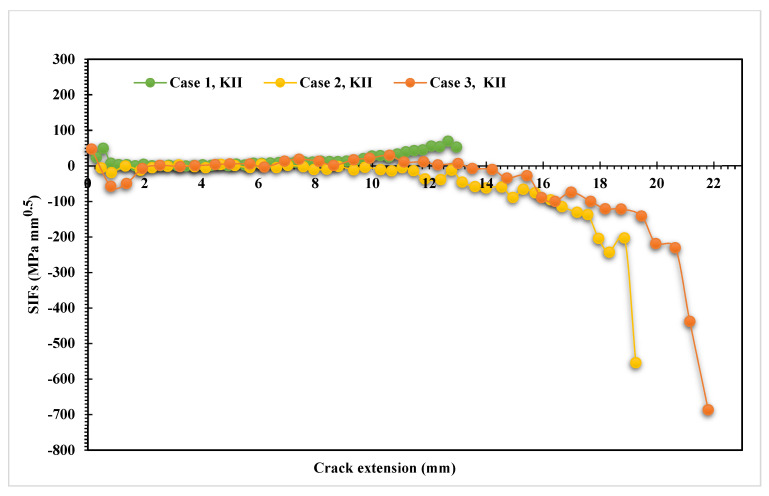
Predicted values of *K_II_* for the three different cases.

The stress contour plot of von Mises stress distribution and maximum principal stress for the three simulated cases are depicted in Figure 12 and Figure 13. As demonstrated in these figures, the higher values of von Mises stresses and the maximum principal stress were observed in case 1, where the upper hole was closer to the crack based on the initial crack position. This is a useful method to see the biggest direction of stress. Both von Mises stress and maximum principal stress are significant factors for crack growth analysis. Figure 14 shows the predicted values of the first mode of SIFs for the three different cases. The first had a maximum value of *K_I_* = 1186 MPmm^0.5^ with a crack length *a* = 12.962 mm, the second had a maximum value of *K_I_* = 4893 MPmm^0.5^ and crack length *a* = 19.263 mm, and the third had a maximum value of *K_I_* = 7143 MPmm^0.5^ and crack length *a* = 21.8 mm. Furthermore, the predicted values of the second mode of SIFs are shown in Figure 15. As shown in this figure for case 1, the values of *K_II_* increase in positive values as the crack grows toward the upper hole in a curvature path, reaching a maximum value of 70 MPmm^0.5^ at the end of the crack path in the hole’s border. In contrast, in cases 2 and 3, the values of *K_II_* increased with a negative value as the crack grew on a curvature trajectory in the opposite direction to case 1, with maximum values of −554 MPmm^0.5^ and −687 MPmm^0.5^, respectively.

**Figure 16:**
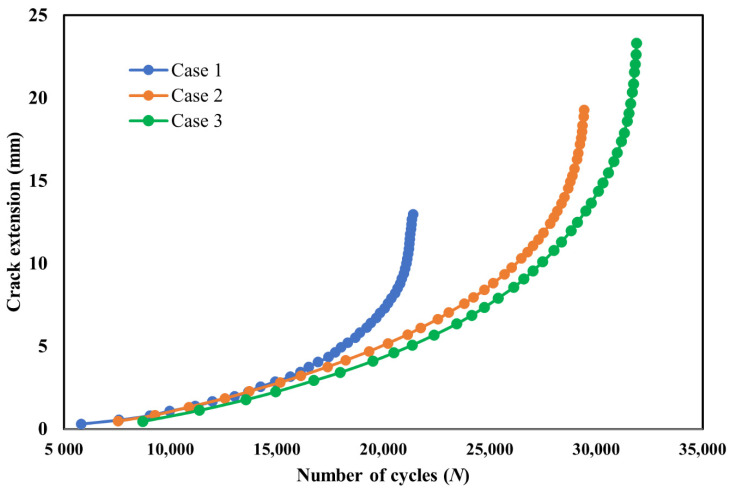
Calculated fatigue life cycles for the three cases.

Fatigue life was calculated with a step-by-step computation of the incremental crack growth based on predicted stress intensity factors. Figure 16 shows the estimated fatigue life cycle all cases; as observed, the number of cycles increases gradually from case 1 to 3 owing to the corresponding increase in the stress intensity factors with the same degree.

### 3.2. Plate Having Circular Hole and Edge Crack with Different Initial Crack-Tip Position

**Figure 17:**
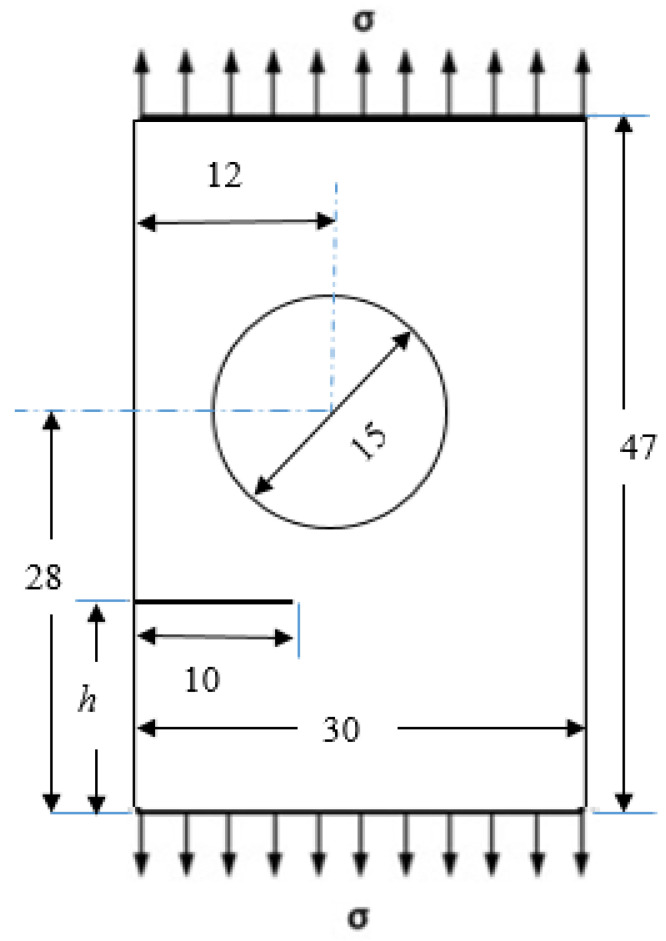
Geometrical dimension for the plate with a circular hole and edge crack (all dimensions in mm).

**Figure 18:**
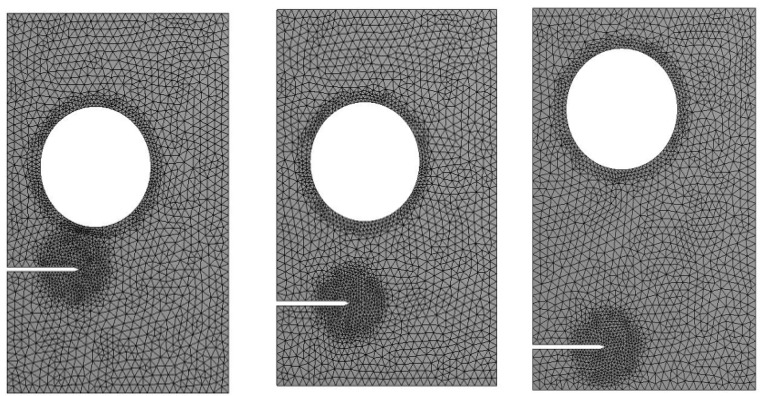
Initial adaptive mesh for the three different configurations of plates with a circular hole and edge crack.

A rectangular plate having a circular hole and edge crack with a different initial crack tip position has been considered. Three alternative pre-crack locations are considered: *h* = 15 mm, *h* = 10 mm, and *h* = 5 mm assuming plane stress state. Figure 17 depicts the geometrical dimensions of this plate, whereas Figure 18 depicts the initial adaptive mesh for these three different alternative configurations. The applied uniaxial stress was *σ* = 20 MPa, elasticity modulus, *E* = 1 GPa, and Poisson’s ratio *ν* = 0.3.

**Figure 19:**
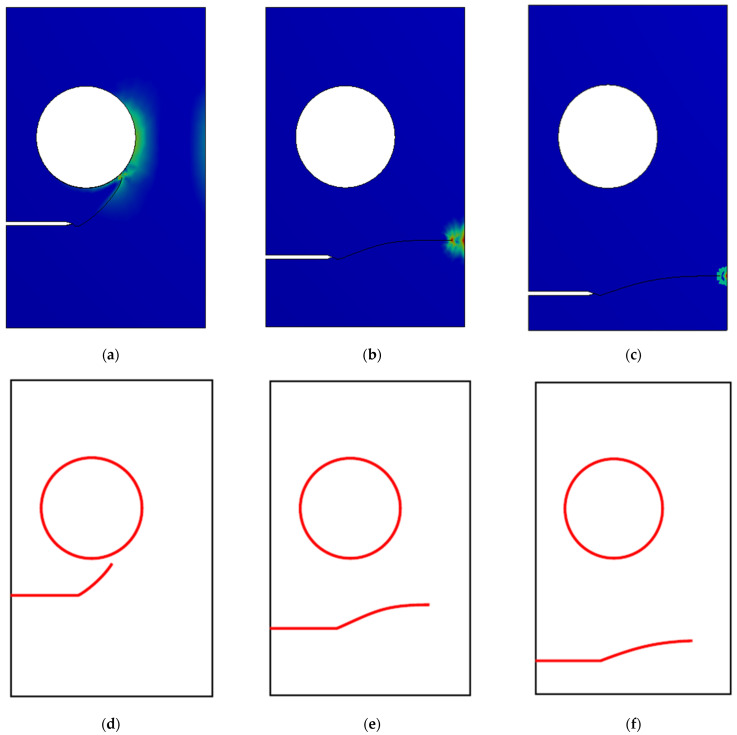
Comparison for the crack growth path, (**a**) *h* = 15 (present study), (**b**) *h* = 10 (present study), (**c**) *h* = 5 (present study), (**d**) *h* = 15 [52], (**e**) *h* = 10 [52], (**f**) *h* = 5. Copyright permission obtained from Elsevier [52].

Mesh refinement is observed executed all over the crack tip and the hole before the crack propagates, and it tends to be carried out only near the crack tip as it grows until it eventually gets more remote from the hole. Figure 19a–c depicted the predicted crack growth trajectories for three different configurations of crack position, which were compared to the numerical results obtained by [52] with a locally refined (LR) B-splines extended isogeometric analysis (XIGA) method, as shown in Figure 19d–f. Essentially, the position of the pre-crack determines the crack trajectory. The research findings of crack trajectories show that the crack grows consistently in the direction of the hole initially with *h* = 15 mm. When the crack approaches the hole, the deviation toward the hole increases until it sinks into the hole. For the second and third configuration, the crack deviates from the initial path at *h* = 10 mm and *h* = 5 mm; then, it grows approximately horizontally to the other edge of the geometry.

**Figure 20:**
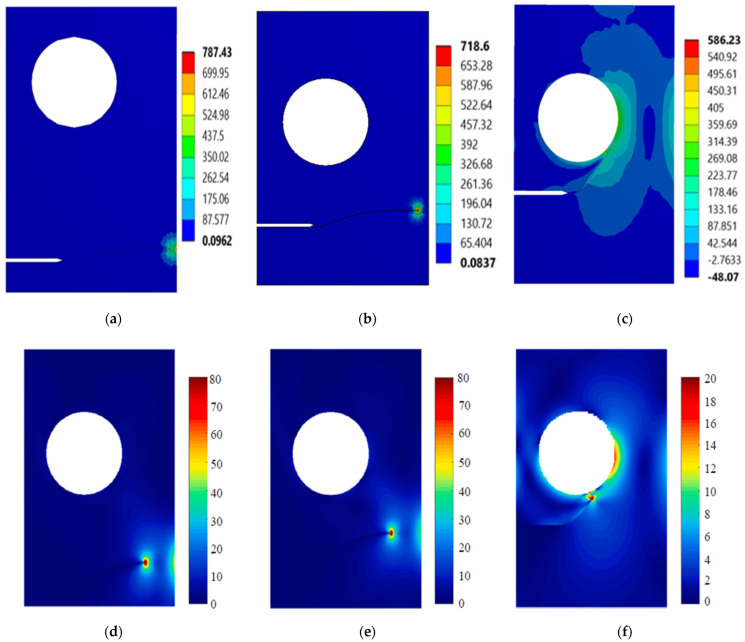
Comparison for the von Mises stress distribution (MPa) (**a**) *h* = 15 (present study), (**b**) *h* = 10 (present study), (**c**) *h* = 5 (present study), (**d**) *h* = 15 [52], (**e**) *h* = 10 [52], (**f**) *h* = 5. Copyright permission obtained from Elsevier [52].

Figure 20 represents von Mises stress distribution at the final step of propagation in comparison to the numerically predicted values obtained by [52]. It is clear that the higher stress concentration occurs mostly in the region of the crack tip for *h* = 5 mm and *h* = 10 mm, and the higher stress concentration occurs mostly near the right side of the hole and the crack tip for *h* = 15 mm. If there was no hole at the plate for mode I loading, the crack would grow in linearly; however, because of the presence of the hole, the linear path was not followed. The crack consistently was fascinated to the hole; either it curves its direction and grows toward it, “sink in the hole”, or it is deflected by it and propagates after missing it “miss the hole”. Overall, the presence of holes in the plate disrupted the stress and strain fields, resulting in interesting curvilinear crack paths on each specimen.

**Figure 21:**
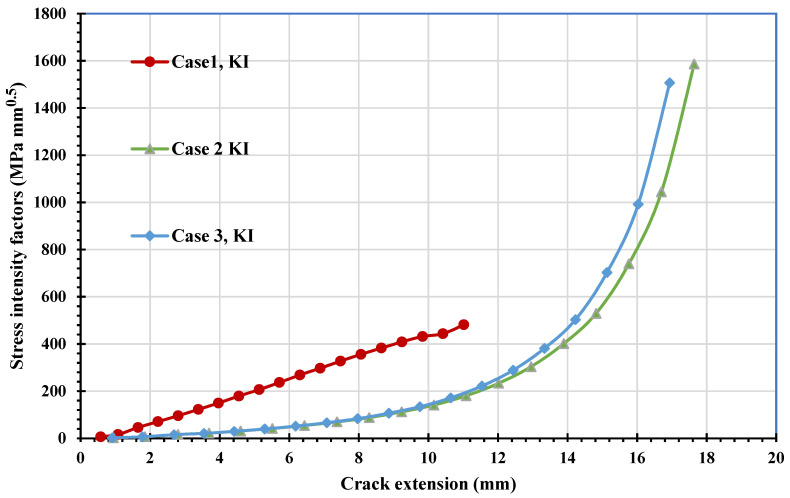
Predicted values of *K_I_* for the three different cases.

**Figure 22:**
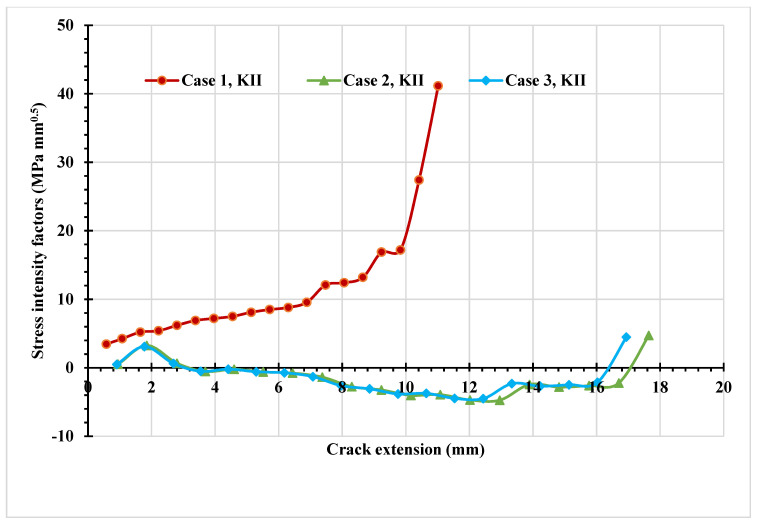
Predicted values of *K_II_* for the three different cases.

The predicted values of the first mode and second mode of SIFs are shown in Figure 21 and Figure 22. The first case displays the greatest *K_I_* and *K_II_* values compared to the other two cases with the same crack length, indicating the effect of the hole as the crack grows toward the hole. The crack will grow in a curved path until it sinks into the hole as the *K_II_* values increase, as seen in case 1. In the second and third cases, there was a slight influence of the hole at the beginning of the crack path, which can also be seen in the increasing of *K_II_*; however, as the *K_I_* values were increased, the crack continued to grow in a straight direction, and this mode dominated the crack growth path with decreasing *K_II_*.

## 4. Conclusions

A crack propagation developed program that hinged on the finite element method was used in probing problems involving holes with different initial crack locations. The stress intensity factors were evaluated using the displacement extrapolation technique, and the angles of crack propagation were computed from maximum circumferential stress theory. The incremental size of the crack during propagation was approximated using the Lagrange interpolation with reference to the stress intensity ratio. A modified Paris law was employed in estimating fatigue life. The crack paths predicted by the developed program align with outcomes from experimental and numerical results in the literature. Influence of the hole on the crack growth path is large when the hole is in close proximity to the crack. Holes act as crack stoppers and attract a crack trajectory to grow. These results reveal the algorithm’s ability to identify crack-stopping holes used in damage tolerance designs. The developed program’s capability to accurately predict the crack path trajectory, stress intensity factors, and fatigue life under constant amplitude loading was demonstrated by this series of simulations.

## Figures and Tables

**Table 1:** Commonly applied $\Delta K_{eq}$ formulas.

Model Provides by Authors	$\Delta K_{eq}\;\mathrm{Expression}$
[45]	ΔKeq=(ΔKI2+2ΔKII2)12
[21]	$\Delta K_{eq}=\sqrt{\Delta K_{1}^{2}+\Delta K_{II}^{2}}$
[46,47]	$\Delta K_{eq}=\frac{\Delta K_{I}}{2}+\frac{1}{2}\sqrt{\Delta K_{I}^{2}+4(1.155\Delta K_{II}^{2})}$
[48]	$\Delta K_{eq}={\left(1.0519\times K_{I}^{4}-0.035\times K_{II}^{4}+2.3056\times K_{I}^{2}\times K_{II}^{2}\right)}^{1/2}$
[37]	$\Delta K_{eq}=\cos\frac{\theta }{2}\left[K_{I}\cos^{2}\frac{\theta }{2}-\frac{3}{2}K_{II}\sin\theta \right]$

## Data Availability

No new data were created or analyzed in this study. Data sharing is not applicable to this article.
